# Interaktive elektronische Visualisierungsformate in der studentischen Ausbildung

**DOI:** 10.1007/s00106-024-01436-9

**Published:** 2024-02-23

**Authors:** Sara M. van Bonn, Jan S. Grajek, Stefanie Rettschlag, Sebastian P. Schraven, Robert Mlynski

**Affiliations:** Klinik und Poliklinik für Hals-Nasen-Ohrenheilkunde, Kopf- und Halschirurgie „Otto Körner“, Universitätsmedizin Rostock, Doberaner Straße 137, 18057 Rostock, Deutschland

**Keywords:** Simulationstraining, Medizinstudierende, Hals-Nasen-Ohren-Heilkunde, Digitale Technologie, Lehre, Simulation training, Medical students, Otorhinolaryngology, Digital technology, Teaching

## Abstract

**Hintergrund:**

Im Rahmen von Kontaktbeschränkungen wird die herkömmliche Lehre derzeit optimierungs- und ausbaufähig. Das Angebot an digitalen Lehrformaten in der studentischen Ausbildung ist sehr heterogen und die Effektivität ungewiss. Diese Studie zielt darauf ab zu untersuchen, inwieweit eine elektronische Visite als Alternative zum herkömmlichen HNO-Anwesenheitspraktikum genutzt werden kann und ob der Einsatz von elektronischen Lehrformaten einen Einfluss auf die Qualität der Lehre ausübt.

**Material und Methoden:**

Anstelle regulärer Anwesenheitspraktika erfolgte einmal wöchentlich der Unterricht am Krankenbett in Echtzeit als Videostream via Tablet. In die prospektive Studie wurden 43 Studierende des 7. Semesters (WS 2020/2021) einbezogen. Mithilfe von Evaluationsbögen wurde der subjektive didaktische Wert verschiedener Visualisierungsformate für die Studierenden untersucht. Vergleichend hinzugezogen wurden die Klausurergebnisse der Vorjahre.

**Ergebnisse:**

Die Mehrheit der Studierenden gaben an, einen Wissenszugewinn durch die elektronische Visite zu haben (93,02 %) und dass diese eine gute Alternative zum herkömmlichen Anwesenheitspraktikum darstellte (69,77 %). Die Qualität der Video- und Audioübertragung sowie Verständlichkeit der gezeigten Fallbeispiele wurde durchgehend als gut bis sehr gut bewertet. Die Klausurergebnisse der Studierenden waren im Testsemester tendenziell leicht schlechter als in den Kontrollsemestern.

**Schlussfolgerungen:**

Die Integration innovativer interaktiver Visualisierungsmöglichkeiten in die Lehre zeigt vielversprechende Perspektiven als Ergänzung zum herkömmlichen Präsenzunterricht. Die Ergebnisse dieser Studie können dazu beitragen, die digitale Lehre weiter auszubauen. Eine Skalierung dieses Modells könnte insbesondere in Ländern mit begrenzter Verfügbarkeit von Präsenzlehrplätzen in Betracht gezogen werden.

## Abstract (en)

**Background:**

In the context of contact restrictions, conventional teaching is currently in need of optimization and expansion. The range of digital teaching formats in student training is very heterogeneous and their effectiveness uncertain. This study aims to investigate the extent to which an electronic ward round can be used as an alternative to the conventional ENT attendance practical course, and whether the use of electronic teaching formats has an influence on the quality of teaching.

**Materials and methods:**

Instead of regular attendance practicals, bedside teaching took place once a week in real time as a video stream via tablet. A total of 43 students in the seventh semester (winter semester 2020/2021) were included in the prospective study. Evaluation forms were used to examine the subjective didactic value of different visualization formats for the students. Examination results from previous years were used for comparison.

**Results:**

The majority of students reported knowledge gain from the electronic rounds (93.02%) and that they were a good alternative to the traditional attendance clerkship (69.77%). The quality of the video and audio transmission as well as the comprehensibility of the case studies presented were consistently rated as good to very good. The students’ examination results tended to be slightly worse in the test group compared to the control students of previous years.

**Conclusion:**

Integration of innovative interactive visualization options into teaching shows promising prospects as a supplement to conventional face-to-face teaching. The results of this study can contribute to the further expansion of digital teaching. Scaling up this model could be considered especially in countries with limited availability of face-to-face teaching.

## Digitale Technologien und Lehrmethoden

Die Vermittlung von Kompetenzen und Wissen hat sich in den letzten Jahren aufgrund der COVID-19-Pandemie drastisch verändert. Die elektronische Umsetzung und erfolgreiche Implementierung erfolgten auf verschiedenen didaktischen Wegen. Aufgrund der Beschränkungen von Präsenzveranstaltungen gewannen computergestützte digitale Formate an Bedeutung und eröffneten zahlreiche neue Möglichkeiten [1–3]. Allerdings stellte die COVID-19-Pandemie die universitären Einrichtungen vor große Herausforderungen. Eine schnelle Umstellung der analogen zur digitalen Lehre war erforderlich. Dies war im medizinischen Alltag, gerade bei den Anwesenheitspraktika, zusätzlich erschwert, da häufig direkte praktische und theoretische Vor-Ort-Unterweisungen durchgeführt wurden [4]. Trotz dieser Herausforderungen konnten innovative Lösungen zur Aufrechterhaltung und Sicherung der studentischen Ausbildung entwickelt werden, wie beispielsweise E‑Learning und Live-Online-Streaming von Vorlesungen oder Operationen. Diese Maßnahmen zeigen nicht nur eine hohe Flexibilität, sondern auch ein beträchtliches innovatives Potenzial in der universitären HNO-Lehre in Deutschland [5–7]. Bei der Testung neuer digitaler Technologien und Lehrmethoden ist es von entscheidender Bedeutung, neben objektiven Verfahren auch den subjektiven Erfolg durch Evaluierungen zu bewerten. Letztere haben vorrangig das Ziel, die Qualität auf Grundlage des erhaltenen Feedbacks zu verbessern, insbesondere im Kontext von Praktika [8–13].

Die Digitalisierung der Lehre hat sich bereits jetzt als ein nützliches Instrument in der medizinischen Ausbildung herausgestellt, da sie v. a. herkömmlichen Lehrmitteln (wie z. B. traditionellen Vorlesungen) nicht unterlegen ist, individualisiert angeboten werden kann und große Flexibilität für die Lernenden ermöglicht [14, 15]. Vorherige Studien haben gezeigt, dass elektronische Lernprogramme und der Einsatz neuartiger Technologien und Visualisierungsformate dazu geführt haben, dass die Studierenden subjektiv zufriedener mit ihren Lernerfahrungen sind. Digitale Tools können vielseitig eingesetzt werden und auch komplexe Lerninhalte vermitteln [1–3, 16]. Zudem zeigten Untersuchungen, dass Studierende in der HNO-Heilkunde auf von Dozenten ausgegebene Materialien als einzige Lernquelle zurückgreifen. Die Bereitstellung digitaler Lehrmethoden und die Anwendung durchdachter digitaler Lehrstrategien bieten die Chance, dem Überfluss an Informationen aus teilweise unklaren Quellen im Internet entgegenzuwirken [17].

Auch die Lehrenden haben durch den Einsatz digitaler Formate die Möglichkeit, bestehende Lehr- und Lernstrukturen neu zu gestalten. Allerdings existieren nur wenige große Studien über die Anwendbarkeit der digitalen Lehre im klinischen studentischen Alltag. Digitale Medien sind weiterhin noch kein flächendeckender Bestandteil der medizinischen Lehre [18, 19].

Ziel dieser Studie war die Evaluation, inwiefern die digitale Transformation der Lehre die Qualität der Lehre beeinflusst. Hierfür wurde den Studierenden anstelle von Präsenzunterricht am Krankenbett, welcher zu diesem Zeitpunkt nicht möglich war, neben den regulären wöchentlichen Online-Vorlesungen, Live-Videostreaming verschiedener Operationen und Online-Lernkursen die Teilnahme an einer elektronischen Visite ermöglicht. Anschließend wurde die elektronische Visite evaluiert und die Klausurergebnisse der Vorjahre miteinander vergleichen.

## Methodik

Während des Wintersemesters 2020/2021 wurde das digitale Lehr- und Lernprogramm der medizinischen Fakultät eines universitären Maximalversorgers ausgeweitet. Die Studie wurde in einem prospektiven Design als Single-Center-Studie durchgeführt. Aufgrund der gemäß Coronaschutzverordnungen der Landesregierung und Weisungen des Rektors der Universität geltenden Einschränkungen für den Präsenzunterricht war das reguläre Anwesenheitspraktikum ausgesetzt. Anstatt dessen wurde den Studierenden, neben regulären wöchentlichen Online-Vorlesungen, Live-Videostreaming von verschiedenen Operationen und Online-Lernkursen, die Teilhabe an einer elektronischen Visite ermöglicht.

Studierende des 7. Semesters, welche für das Blockpraktikum im Wintersemesters 2020/2021 eingeteilt waren, wurden über das Projekt informiert und über die Teilnahme an der anschließenden freiwilligen Evaluation aufgeklärt. Die Studierenden konnten nach Erhalt eines individuellen und wöchentlich wechselnden Passworts von jedem Ort in Deutschland sowie weltweit über Zoom (Fa. Zoom Video Communications Inc., San Jose, CA, USA) auf den Stream zugreifen und an der elektronischen Visite teilhaben. Pro Gruppe nahmen 7–10 Studierende an der elektronischen Visite teil. Der digitale Unterricht am Krankenbett erfolgte einmal wöchentlich für jeweils eine Gruppe zu einem vorher festgelegten Termin. Die Gruppen wechselten wöchentlich. Der Mentor/die Mentorin hat die Zoom-App auf einem iPad der 9. Generation (Fa. Apple Inc., Cupertino, CA, USA) genutzt und mit diesem gefilmt. Der Inhalt der etwa 60 min langen elektronischen Visite richtete sich nach den unterschiedlichen Krankheitsbildern der Patienten, welche zu diesem Zeitpunkt stationär waren. Je nach Krankheitsbild der Patienten erfolgte die Patientenvorstellung durch den jeweiligen Arzt/die jeweilige Ärztin bzw. wurde auch selber von den Studierenden in direkter Interaktion mit den Patienten erhoben. Die klinische Untersuchung bzw. der Verbandswechsel erfolgte durch den Arzt/die Ärztin. Klinisch interessante Befunde wurde mit einer Kamera am Ohrmikroskop bzw. an den Endoskopen aufgenommen und den Studierenden auf einem Bildschirm gezeigt. Vor der elektronischen Visite gaben die Patienten ihr schriftliches Einverständnis für die anonymisierte Übertragung von Anamnese sowie Krankheitsbildern zu Ausbildungszwecken. Die Studierenden konnten zwischendurch zu jeder Zeit Fragen stellen oder die Chatfunktion nutzen, sodass sie vom gesamten Publikum diskutiert werden konnten. Die elektronische Visite erfolgte datenschutzkonform.

Nach Ende der elektronischen Visite wurden die Studierenden per E‑Mail informiert und gebeten, einen Bewertungsfragebogen auszufüllen, der auf der Online-Plattform ILIAS (ILIAS open-source e‑Learning e. V., Köln, Deutschland) bereitgestellt wurde (Tab. 1). Das Open-Source-Produkt ILIAS ist ein freies Hypertext-Transfer-Protokoll, das für die internetbasierte Bereitstellung von Lehr- und Lernmaterialien genutzt wird. Die ersten 12 Fragen waren auf einer 5‑stufigen Likert-Skala zu beantworten (trifft zu = 6 Punkte; trifft eher zu = 5 Punkte; trifft teilweise = 4 Punkte; trifft eher nicht zu = 3 Punkte; trifft nicht zu = 2 Punkte, kann ich nicht beantworten = 1 Punkt), 5 Aussagen waren anhand einer Ratingskala mit Abstufungen von sehr gut (1) bis ungenügend (6) zu bewerten und 2 Fragen waren offen zu beantworten. Eine Evaluation der die durchführenden Ärzte/Ärztinnen erfolgte nicht.

**Table 1:** 

*Evaluationsbogen: Antwortmöglichkeiten: „trifft zu“, „trifft eher zu“, „trifft teilweise zu“, „trifft eher nicht zu“, „trifft nicht zu“ und die Option „kann ich nicht beantworten“*	
1.	Die E‑Visite brachte mir einen großen subjektiven Wissenszugewinn.
2.	Die E‑Visite bietet eine gute Alternative zum herkömmlichen Anwesenheitspraktikum.
3.	Die Möglichkeit zur E‑Visite sollte vermehrt angeboten werden.
4.	Meine zur Verfügung stehende Hardware (Tablet, PC, Handy etc.) konnte die E‑Visite problemlos abspielen.
5.	Das Live-Chat-Modul empfinde ich als eine sinnvolle Möglichkeit zur Interaktion mit dem Arzt.
6.	Eine direkte Interaktion ist während der E‑Visite möglich.
7.	Ich wäre bereit, für die dauerhafte mögliche Teilnahme an der E‑Visite Geld zu zahlen.
8.	Die Teilnahme an der E‑Visite von zu Hause aus ermöglicht mir, meinen Studienplan flexibler zur gestalten.
9.	Das Fach HNO-Heilkunde eignet sich gut für den Einsatz einer E‑Visite.
10.	Die studentische Ausbildung in der HNO-Heilkunde setzt einen direkten (vor Ort) Arzt-Studierenden-Kontakt voraus.
11.	Ich wünsche mir im Allgemeinen ein größeres Angebot für elektronische Lernformate.
12.	Ich würde gerne, auch zu einem späteren Zeitpunkt, erneut auf die Daten der E‑Visite zurückgreifen.
*Bewertungsskala: Antwortmöglichkeiten „sehr gut“ (1), „gut“ (2), „befriedigend“ (3), „ausreichend“ (4), „nicht ausreichend“ (5), „mangelhaft“ (6)*	
13.	Qualität der Videoübertragung
14.	Qualität der Audioübertragung
15.	Stabilität der Verbindung zum Channel
16.	Verständlichkeit der gezeigten Fallbeispiele
17.	Interaktionsmöglichkeit mit dem Arzt
*Offene Fragen*	
18.	Ich habe für die E‑Visite folgende Hardware genutzt: bspw. Mobiltelefon, Tablet, PC, Laptop
19.	Zum Streamen habe ich folgenden Browser genutzt: bspw. Firefox, Opera, MS-Explorer, Safari, Google Chrome

Wie in den Jahren zuvor wurde zum Abschluss des Semesters eine Verbundklausur geschrieben. Die Ergebnisse des Wintersemesters 2020/2021 wurden mit den Ergebnissen der Vorjahre, in welchen die Lehre ohne Kontaktbeschränkungen regulär vollzogen wurde, verglichen. Die Bewertung der Klausuren ist anhand von Schulnoten erfolgt (sehr gut – 1, gut – 2, befriedigend – 3, ausreichend – 4, mangelhaft – 5, ungenügend – 6).

Die statistischen Analysen wurden mit Prism (Version 8, GraphPad Software, La Jolla, CA, USA) durchgeführt. Es wurde der Mann Whitney-Test durchgeführt. Das Signifikanzniveau wurde auf *p* < 0,05 festgesetzt. Wenn nicht anders angegeben, werden die Daten als Mittelwerte mit Standardabweichung (SD) oder als absolute Werte mit Prozenten angegeben.

## Ergebnisse

**Figure 1:**
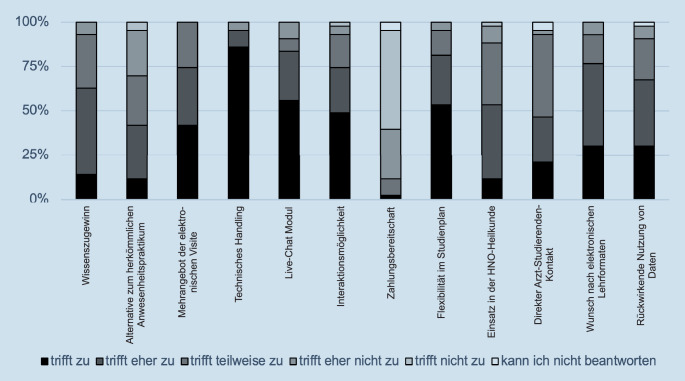

Von im Wintersemester 2020/2021 242 eingeschriebenen Studierenden waren 102 zur Teilnahme am Praktikum in der HNO verpflichtet. Von den 102 Studierenden, welche an der elektronischen Visite teilnahmen, füllten nur 43 Studierende den Evaluationsbogen (Tab. 1) vollständig aus. Somit lag die Rücklaufquote nur bei 42,16 %. Die Antworten der Studierenden auf den Evaluationsbogen sind in Abb. 1 und 2 dargestellt.

**Figure 2:**
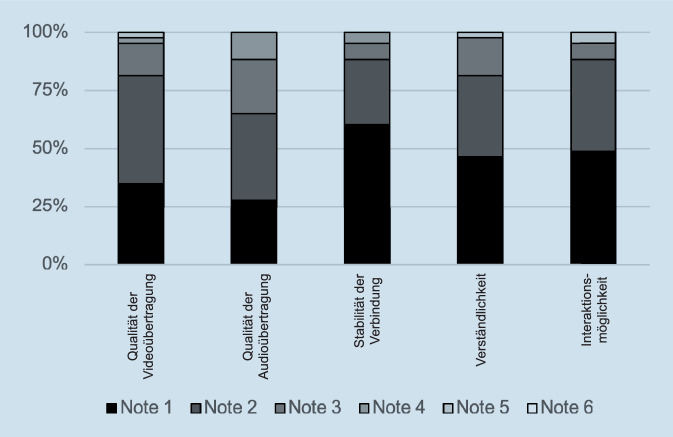

### Wissenszugewinn

Der Aussage, dass die E‑Visite einen großen subjektiven Wissenszugewinn brachte, stimmten 6 Teilnehmer (13,95 %) voll und ganz zu (6 Punkte). Dieser Aussage stimmten 21 Teilnehmer (48,84 %) zu (5 Punkte), und 13 Teilnehmer (30,23 %) waren unentschlossen (4 Punkte).

### Alternative zum herkömmlichen Anwesenheitspraktikum

Der Aussage, dass die die E‑Visite eine gute Alternative zum herkömmlichen Anwesenheitspraktikum ist, stimmten 5 Teilnehmer (11,63 %) voll und ganz zu (6 Punkte). Dieser Aussage stimmten 13 Teilnehmer (30,23 %) zu (5 Punkte), 12 Teilnehmer (27,91 %) waren unentschlossen (4 Punkte), 11 Teilnehmer (25,58 %) stimmten dieser Aussage eher nicht zu (3 Punkte), und 2 Teilnehmer (4,65 %) stimmten dieser Aussage nicht zu (2 Punkte).

### Mehrangebot der elektronischen Visite

Der Aussage, dass die Möglichkeit zur E‑Visite vermehrt angeboten werden sollte, stimmten 18 Teilnehmer (41,86 %) voll und ganz zu (6 Punkte). Dieser Aussage stimmten 14 Teilnehmer (32,56 %) zu (5 Punkte), und 11 Teilnehmer (25,58 %) waren unentschlossen (4 Punkte).

### Technisches Handling

Der Aussage, dass die ihnen zur Verfügung stehende Hardware die E‑Visite problemlos abspielen konnte, stimmten 37 Teilnehmer (86,05 %) voll und ganz zu (6 Punkte). Dieser Aussage stimmten 4 Teilnehmer (9,30 %) zu (5 Punkte), und 2 Teilnehmer (4,65 %) stimmten dieser Aussage eher nicht zu (3 Punkte).

### Live-Chat-Modul

Der Aussage, dass das Live-Chat Modul als eine sinnvolle Möglichkeit zur Interaktion mit dem Arzt empfunden wurde, stimmten 24 Teilnehmer (55,81 %) voll und ganz zu (6 Punkte). Dieser Aussage stimmten 12 Teilnehmer (27,91 %) zu (5 Punkte), 3 Teilnehmer (6,98 %) waren unentschlossen (4 Punkte), und 4 Teilnehmer (9,30 %) stimmten dieser Aussage eher nicht zu (3 Punkte).

### Interaktionsmöglichkeit

Der Aussage, dass eine direkte Interaktion während der E‑Visite möglich ist, stimmten 21 Teilnehmer (44,84 %) voll und ganz zu (6 Punkte). Dieser Aussage stimmten 11 Teilnehmer (22,58 %) zu (5 Punkte), 8 Teilnehmer (18,60 %) waren unentschlossen (4 Punkte), 2 Teilnehmer (4,65 %) stimmten dieser Aussage eher nicht zu (3 Punkte), und ein Teilnehmer (2,33 %) stimmte der Aussage nicht zu (2 Punkte).

### Zahlungsbereitschaft

Ein Teilnehmer (2,33 %) stimmten der Aussage voll und ganz zu (6 Punkte), dass die Bereitschaft, für eine dauerhafte Teilnahme an der E‑Visite Geld zu zahlen, da ist, 4 Teilnehmer (9,30 %) waren unentschlossen (4 Punkte), 12 Teilnehmer (17,91 %) stimmten dieser Aussage eher nicht zu (3 Punkte), 24 Teilnehmer (55,81 %) stimmten der Aussage nicht zu (2 Punkte), und 2 Teilnehmer (4,65 %) konnten keine Aussage treffen (1 Punkt).

### Flexibilität im Studienplan

Der Aussage, dass die Teilnahme an der E‑Visite von zu Hause aus es ermöglicht, den Studienplan flexibler zu gestalten, stimmten 23 Teilnehmer (53,49 %) voll und ganz zu (6 Punkte). Dieser Aussage stimmten 12 Teilnehmer (27,91 %) zu (5 Punkte), 6 Teilnehmer (13,95 %) waren unentschlossen (4 Punkte), und 2 Teilnehmer (4,65 %) stimmten dieser Aussage eher nicht zu (3 Punkte).

### Einsatz in der HNO-Heilkunde

Der Aussage, dass das Fach HNO-Heilkunde gut geeignet ist für den Einsatz einer E‑Visite, stimmten 5 Teilnehmer (11,63 %) voll und ganz zu (6 Punkte). Dieser Aussage stimmten 18 Teilnehmer (41,86 %) zu (5 Punkte), 15 Teilnehmer (34,88 %) waren unentschlossen (4 Punkte), 4 Teilnehmer (9,3 %) stimmten dieser Aussage eher nicht zu (3 Punkte), ein Teilnehmer (2,33 %) stimmte dieser Aussage nicht zu (2 Punkte).

### Direkter Arzt-Studierenden-Kontakt

Der Aussage, dass die studentische Ausbildung in der HNO-Heilkunde einen direkten (vor Ort) Arzt-Studierenden-Kontakt voraussetzt, stimmten 9 Teilnehmer (20,93 %) voll und ganz zu (6 Punkte). Dieser Aussage stimmten 11 Teilnehmer (25,58 %) zu (5 Punkte), 20 Teilnehmer (45,51 %) waren unentschlossen (4 Punkte), ein Teilnehmer (2,33 %) stimmten dieser Aussage eher nicht zu (3 Punkte), und 2 Teilnehmer (4,65 %) konnten keine Aussage treffen (1 Punkt).

### Wunsch nach elektronischen Lernformaten

Der Aussage, dass ein größeres Angebot für elektronische Lernformate gewünscht ist, stimmten 13 Teilnehmer (30,23 %) voll und ganz zu (6 Punkte). Dieser Aussage zu (5 Punkte) stimmten 20 Teilnehmer (45,51 %), 7 Teilnehmer (16,28 %) waren unentschlossen (4 Punkte), und 3 Teilnehmer (6,98 %) stimmten dieser Aussage eher nicht zu (3 Punkte).

### Rückwirkende Nutzung von Daten

Der Aussage, dass das Zurückgreifen auf die Daten der E‑Visite zu einem späteren Zeitpunkt gewünscht ist, stimmten 13 Teilnehmer (30,23 %) voll und ganz zu (6 Punkte). Dieser Aussage stimmten 16 Teilnehmer (37,21 %) zu (5 Punkte), 10 Teilnehmer (23,26 %) waren unentschlossen (4 Punkte), 3 Teilnehmer (6,98 %) stimmten dieser Aussage eher nicht zu (3 Punkte), und ein Teilnehmer (2,33 %) konnte keine Aussage treffen (1 Punkt).

### Qualitätskriterien

Die Qualität der Videoübertragung bewerteten 35 Teilnehmer (81,39 %) mit gut bis sehr gut. Die Qualität der Audioübertragung bewerteten 28 Teilnehmer (65,12 %) mit gut bis sehr gut. Die Stabilität der Verbindung zum Channel bewerteten 38 Teilnehmer (88,38 %) mit gut bis sehr gut. Die Verständlichkeit der gezeigten Fallbeispiele bewerteten 35 Teilnehmer (81,39 %) mit gut bis sehr gut. Die Interaktionsmöglichkeit mit dem Arzt bewerteten 38 Teilnehmer (88,37 %) mit gut bis sehr gut.

### Hardware

Zur Teilnahme an der Live-Operation nutzten 34 Studierende (79,07 %) einen Laptop, ein Studierender (2,33 %) ein Tablet, 6 Studierende (13,95 %) einen Desktop Computer und 2 Studierende (4,65 %) ein Mobiltelefon. Als Internetbrowser verwendeten 16 Studierende (37,21 %) Google Chrome, 11 Studierende (25,58 %) Safari, 13 Studierende (30,23 %) Firefox, 2 Studierende (4,65 %) Microsoft Edge und ein Studierender (2,33 %) Opera.

### Klausurergebnisse

Es zeigte sich im Wintersemester 2020/2021, dass 39,6 % der Studierenden die Note 1 und 2 erhalten haben. In den Jahren zuvor waren es 65,92 % (2017/2018), 52,81 % (2018/2019) und 68,89 % (2019/2020). Im Wintersemester 2017/2018 (*n* = 226) haben 42 Studierende die Note 1, 107 Studierende die Note 2, 65 Studierende die Note 3 und 11 Studierende die Note 4 erhalten. Im Wintersemester 2018/2019 (*n* = 231) haben 13 Studierende die Note 1, 109 Studierende die Note 2, 91 Studierende die Note 3 und 16 Studierende die Note 4 erhalten. Im Wintersemester 2019/2020 (*n* = 209) haben 44 Studierende die Note 1, 100 Studierende die Note 2, 51 Studierende die Note 3 und 13 Studierende die Note 4 erhalten. Im Wintersemester 2020/2021 (*n* = 242) haben 17 Studierende die Note 1, 79 Studierende die Note 2, 93 Studierende die Note 3 und 37 Studierende die Note 4 erhalten. Der Gesamtdurchschnitt der Klausurergebnisse lag 2017/2018 bei 2,21; 2018/2019 bei 2,5; 2019/2020 bei 2,17 und 2020/2021 bei 2,81. Insgesamt sind in den 4 Jahren 20 Studierende durchgefallen (16 Studierende davon im Wintersemester 2020/2021).

**Figure 3:**
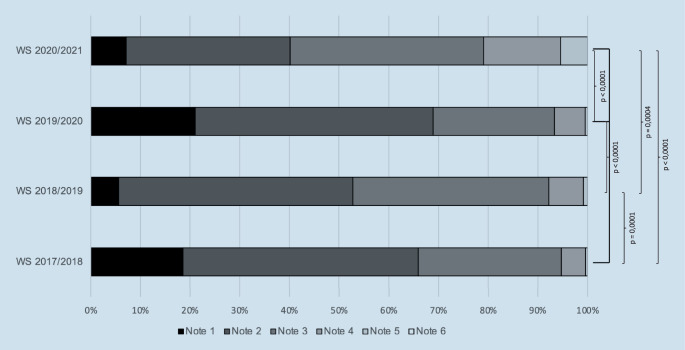

Die Klausurergebnisse des Wintersemesters 2020/2021 sind im Durchschnitt signifikant schlechter im Vergleich zu den Vorjahren mit Präsenslehre (2019/2020: *p* < 0,0001; 2018/2019: *p* = 0,0004; 2017/2018: *p* < 0,0001). Zudem zeigte sich die Benotung im Wintersemester 2018/2019 im Durchschnitt signifikant schlechter im Vergleich zu 2019/2020 (*p* < 0,0001) und 2017/2018 (*p* = 0,0001). Die deskriptive Statistik ist in Tab. 2 und die Auflistung der Klausurergebnisse ist Abb. 3 zu entnehmen.

**Table 2:** 

Aussagen	WS 2017/18	WS 2018/19	WS 2019/20	WS 2020/21
Studierende (*n*)	226	231	209	242
Minimum	1	1	1	1
25%-Perzentile	2	2	2	2
Median	2	2	2	3
75%-Perzentile	3	3	3	3
Maximum	5	5	5	5
Spannweite	4	4	4	4
Mittelwert	2,21	2,5	2,172	2,82
Standardabweichung	0,82	0,75	0,85	0,99

## Diskussion

Durch den Einsatz neuer Bildungstrends im Rahmen eines raschen technologischen Wandels entwickelt sich die Gesundheitsversorgung, aber auch die medizinische Aus- und Weiterbildung stetig fort. Vorangetrieben u. a. durch die COVID-19-Pandemie wurde die Umstellung der analogen auf digitale Lehre und somit die Integration von Zukunftstrends in der medizinischen Ausbildung. Aufgrund von Einschränkungen des Präsenzunterrichts mussten zunächst aus der Not heraus zu Beginn der Pandemie schnell digitale Lehrangebote geschaffen werden [11, 19, 20]. Doch haben digitale Lehrangebote den gleichen pädagogischen Wert wie Präsenzformate?

### Lernumfeld

Die Studierenden sind in einer Welt mit digitalem Einfluss aufgewachsen. Sie sind anders vernetzt als frühere Generationen und haben ein anderes Verständnis für die Integration von digitalen Medien im Sinne eines kontextbezogenen und angewandten Lernens [21, 22]. Außerdem hat sich auch das Lernumfeld gewandelt. Künftige Ärzte werden mit komplexeren Gesundheitsproblemen in der Gesellschaft und heterogeneren Patientengruppen konfrontiert sein [23, 24]. Viele erwerben ihre Erfahrungen im Bereich des passiven digitalen Lernens durch das wiederholte Absolvieren von Online-Prüfungen, wobei das Prinzip „assessment drives learning“ Anwendung findet [25]. Die herkömmliche Art der Wissensaneignung durch Lehrbücher und bibliothekarisches Lernen ist veraltet und zudem stark von Selbstmotivation und Lernfähigkeit abhängig. Da die HNO-Heilkunde ein im Lehrplan eher unterrepräsentiertes Fach darstellt, soll durch den Einsatz neuer Visualisierungsmethoden ein Lernanreiz geschafften und das Interesse gesteigert werden. Denn besonders aufgrund fortschrittlicher Visualisierungsmöglichkeiten ist das Fachgebiet der HNO eine ausgezeichnete Grundlage für die Entwicklung neuer Bildungsressourcen [5, 7].

Aktuelle verfügbare Angebote digitaler Lehr- und Lernformate sind sehr heterogen und kommen im Studienalltag bisher nur punktuell zum Einsatz. Häufig stehen den Studierenden klassische Formate, wie z. B. E‑Learning-Tools zu Verfügung [25]. Weitere interaktive audio- und videobasierte Modelle mit unterschiedlichen Visualisierungsmöglichkeiten sind stark hinterfragt und würden bestehende Lehrmethoden unterstützen [7]. Nachweislich steigert die Anwendung digitaler Medien die Motivation und bietet somit die Möglichkeit, das Verständnis für die Patientenversorgung zu vertiefen [21, 22]. Die subjektive Wahrnehmung des Lernprozesses gilt als entscheidender Indikator für den individuellen Lernerfolg [2, 26].

### Wissensvermittlung

Die vorliegende Studie zeigt, dass ein Großteil der Studierenden (62,79 %) einen subjektiven Wissenszugewinn empfinden und sich einen vermehrten Einsatz digitaler Lehrmethoden wünschen (74,42 %). Die direkte Interaktion mit dem Arzt konnte während der elektronischen Visite problemlos erfolgen (83,72 %), und auch das technische Handling war für die Studierenden mehr als zufriedenstellend (95,35 %). Der Datenschutz darf allerdings nicht außer Acht gelassen werden, denn die Plattform für die virtuelle Visite muss eine sichere Verschlüsselungstechnologie verwendet werden, um die Vertraulichkeit von Patientendaten zu gewährleisten. Besonders die örtliche Ungebundenheit und Flexibilität im Stundenplan (81,4 %) empfanden die Studierenden als angenehm.

Neben regulären wöchentlichen Online-Vorlesungen, Live-Videostreaming verschiedener Operationen und Online-Lernkursen über die Lernplattform ILIAS nahmen die Studierenden an der elektronischen Visite teil [1]. Zur objektiven Beurteilung der Effektivität wurden die Klausurergebnisse der Vorjahre miteinander verglichen. Die gestellten Multiple-Choice-Fragen waren vom Anforderungsniveau in den 4 Jahren in etwa gleich. Zudem war der Ersteller der Klausuren in den Wintersemestern 2017/2018 bis 2020/2021 ein und dieselbe Person, sodass hierdurch auch von einer besseren Vergleichbarkeit auszugehen ist. Die Benotung im Wintersemester 2020/2021 ist statistisch signifikant schlechter ausgefallen als bei den Kontrollgruppen in den vorangegangenen Jahren mit Präsenzlehre. Allerdings ist auch das Wintersemester 2018/2019 statistisch im Vergleich zum Wintersemester 2017/2018 und 2019/2020 schlechter ausgefallen. Im Vergleich kann somit eventuell von einer normalen Fluktuation ausgegangen werden. Die Umstellung auf die digitale Lehre ist somit nicht die einzige veränderte Variable im Vergleich zu den Vorjahren gewesen. Die Umstände haben sich sowohl global als auch vor Ort massiv geändert und sind mit erheblichen Einschränkungen der privaten und studentischen Bereiche einhergegangen (wie bspw. das Schreiben der Klausur unter kontakteingeschränkten Bedingungen). Die Ergebnisse lassen vermuten, dass die digitale Umsetzung der Lehre zwar die fehlende, v. a. praktische Ausbildung gut überbrücken kann und ein innovatives Lerninstrument darstellt, die herkömmliche Lehre, insbesondere Vor-Ort-Unterweisungen aber nicht ersetzt. Die Umstellung auf elektronische Lehrformate hatten keinen massiven negativen didaktischen Einfluss auf die Qualität der Lehre. Eine Fortführung der Studie wird zeigen, ob die Ergebnisse ein jahrgangsspezifisches Problem oder ein Problem des Lehrformats ist.

Grundsätzlich zielte die interaktive elektronische Visite als Ersatz für das herkömmliche Blockpraktikum darauf ab, die Anamneseführung, somit Grundlagen der Befunderhebung, und verschiedene Krankheitsbilder kennenzulernen. Ein praktisches Erlernen von Arztgesprächen, die direkte Wahrnehmung emotionaler Regungen des Patienten während der Interaktion und der Körperkontakt bei der klinischen Untersuchung von Patienten kann so nur bedingt trainiert werden. In der virtuellen Visite wurden den Studierenden Untersuchungstechniken erläutert und demonstriert, konnten jedoch nur auf theoretischer Ebene vermittelt werden. Ziel der elektronischen Visite war es somit nicht, praktische Fertigkeiten im Sinne von HNO-spezifischen Untersuchungstechniken zu üben und zu festigen. Die Handhabung und das Üben der Untersuchungstechniken erfolgte bereits einige Semester vorher im Untersuchungskurs. Die Durchführung von HNO-spezifischen Untersuchungstechniken erfordert ein gewisses Maß an praktischen Fertigkeiten, welches nur durch mehrfaches Wiederholen gegeben und sehr erfahrungsabhängig ist [27]. Trotz des fehlenden praktischen Anteils sahen 41,86 % der Studierenden die elektronische Visite als eine gute Alternative zum Anwesenheitspraktikum.

### Vermittlung praktischer Fähigkeiten

Eine Überprüfung praktischer Fähigkeiten z. B. im Rahmen der mündlich-praktischen Abschlussprüfung OSCE (Objective Structured Clinical Examination) war nicht möglich, da diese aufgrund der bestehenden Kontakteinschränkungen nicht erfolgen durfte. Zudem fokussiert derzeit die OSCE inhaltlich auf die „großen“ Fachbereiche (Notfallmedizin, innere Medizin und Allgemeinchirurgie). Die HNO-Heilkunde ist lediglich ein kleiner Teil und kann somit aufgrund der geringen Präsenz des Fachbereichs nur eingeschränkt zur Beurteilung klinisch praktischer Fähigkeiten herangezogen werden. Es existieren allerdings Untersuchungen, welche belegen, dass praktische Fähigkeiten, nach entsprechender sorgfältiger Planung und Strukturierung, auch im Rahmen von digitalen Lehrveranstaltungen effektiv vermittelt werden können [28]. Zweifel bleiben diesbezüglich hinsichtlich der Effektivität, da u. a. das klinische Setting fehlt und zumeist die passenden Untersuchungsmaterialien nicht vorhanden sind.

Ein bekanntes Hindernis für die Umsetzung digitaler Lehr- und Lernformate war bisher der erhöhte Zeitaufwand, welcher für die Erstellung digitaler Materialien notwendig war, und die mangelnde technische Ausstattung [26, 29]. In dieser Studie gab der für die elektronische Visite verantwortliche Arzt/Ärztin keinen relevanten Mehraufwand für den studentischen Unterricht im Arbeitsalltag im Vergleich zum herkömmlichen Anwesenheitspraktikum an. Tatsächlich können durch einen Arzt/eine Ärztin in derselben Zeit mehr Studierende betreut werden. Diese Skalierung des Unterrichts stellt eine Alternative bei knappen Ressourcen und/oder Personalmangel dar. Zudem ist die Umsetzung des elektronischen Lehrformats mit einfachen verfügbaren Mitteln in vernünftiger Qualität gegeben, wie Aussagen 13–17 zeigten.

### Evaluation

Limitierend für diese Studie ist, dass die Teilnahme an der Evaluation auf freiwilliger Basis erfolgte und somit die Teilnehmerquote nur bei 42,16 % lag. Es besteht deshalb die Möglichkeit einer Verzerrung des Ergebnisses, da die Motivation für die Teilnahme nicht erfasst wurde. Es kann nicht ausgeschlossen werden, dass lediglich Studierende mit hoher Motivation ein Feedback gegeben haben [12]. Zukünftige Studien sollten deshalb die Stichprobengröße besser berücksichtigen und Fragen zur Teilnahmemotivation inkludieren. Durch die Einschränkungen der Präsenzlehre im Wintersemester 2020/2021 konnte die subjektive Evaluation nur additiv, wöchentlich bei einer Gruppe an Studierenden, geprüft werden. Eine Fortsetzung der elektronischen Visite erfolgte phasenweise, da in den folgenden Jahren teilweise die Einschränkungen für den Präsenzunterricht aufgehoben wurden. Geeignete Daten aus den Vorjahren zum Vergleich waren naturgemäß nicht erhoben worden. Eine objektive Überprüfung des Lernerfolgs war deshalb an die Auswertung der Klausurergebnisse gekoppelt. Die Auswertung der Klausuren kann als Ergänzung zu dem subjektiven Evaluationsbogen allerdings nur bedingt genutzt werden, da lediglich ein Teil des Semesters (102 Studierende) das Praktikum elektronisch absolviert hat. Somit war der andere Teil des Semesters (137 Studierende) auf die regulären wöchentlichen Online-Vorlesungen, Live-Videostreaming verschiedener Operationen und Selbststudium durch Online-Lernkurse über die Lernplattform ILIAS angewiesen. Hierdurch kommt es zu einer Verzerrung und eingeschränkten Beurteilbarkeit der Ergebnisse. Der Vergleich mit den Klausurergebnissen sollte daher eher als richtungsweisend gewertet werden.

Eine qualitative und quantitative Evaluation neu implementierter Formate ist essenziell, da die Strategien der digitalen Lehre ein sich schnell wandelnder und steter Anpassungsprozess sind und auf technologischen Fortschritten beruhen [17, 30]. In strukturschwachen Ländern mit eingeschränktem Angebot für Präsenzunterricht dürften skalierbare, strukturierte, digitale Lehrkonzepte zudem von besonderer Bedeutung sein. Generell kann die Implementierung neuer digitaler Lehrformate die herkömmliche Lehre ergänzen und sollte daher generell in das Lehrkonzept integriert werden. Der Patientenkontakt und auch die Vermittlung praktischer Fähigkeiten können hierdurch allerdings nicht ersetzt werden.

## Fazit für die Praxis

- Die Verwendung neuer interaktiver Visualisierungsmöglichkeiten in der Lehre stellt einen vielversprechenden Ansatz als Alternative (insbesondere in Zeiten der Pandemie) oder Ergänzung zum traditionellen Präsenzunterricht dar.
- Ein Ausbau der digitaler Lehr- und Lernkonzepte kann auf Basis dieser Studie unterstützt werden.
- Eine weitere Förderung und systematische Aufarbeitung von Erfahrungen mit dem Einsatz digitaler interaktiver Lehr- und Lernkonzepte ist erforderlich, um grundlegende Möglichkeiten von innovativen Neuerungen im Lehrplan und dem gezielten Einsatz virtueller Elemente zu implementieren.
- Hybride Formate stellen die Zukunft dar.
- In strukturschwachen Ländern mit eingeschränktem Angebot für Präsenzunterricht dürften skalierbare, strukturierte, digitale Lehrkonzepte von besonderer Bedeutung sein.
